# FW-PSO Algorithm to Enhance the Invulnerability of Industrial Wireless Sensor Networks Topology

**DOI:** 10.3390/s20041114

**Published:** 2020-02-18

**Authors:** Ying Zhang, Guangyuan Yang, Bin Zhang

**Affiliations:** College of Information Engineering, Shanghai Maritime University, Shanghai 201306, China; yangguangyuan56@stu.shmtu.edu.cn (G.Y.); zhangbin@shmtu.edu.cn (B.Z.)

**Keywords:** FW-PSO algorithm, industrial wireless sensor networks, invulnerability, scale-free, topology optimization

## Abstract

When an industrial wireless sensor network (WSN) is seriously disturbed and intentionally attacked, sometimes it fails easily, even leading to the paralysis of the entire industrial wireless network. In order to improve the invulnerability of networks, in this paper, the scale-free network in complex networks is taken as the research object, and the industrial WSN with scale-free characteristics is modeled. Based on the advantages of the fireworks algorithm, such as strong searching ability and diversity of population, a so-called fireworks and particle swarm optimization (FW-PSO) algorithm is proposed, which can improve the global search ability and convergence speed effectively. The proposed FW-PSO algorithm is used to optimize the network topology and form a network with the largest natural connectivity, which can effectively promote the ability of network to resist the cascade failure problem. The dynamic invulnerability of the optimized network under highest-degree (HD) attack and lowest-degree (LD) attack strategies, as well as the static invulnerability under random attack, were evaluated respectively. Simulation experiments show that the industrial WSN optimized by FW-PSO can significantly improve the performance of the dynamic and static invulnerabilities compared with the initial network and the networks optimized by the other two existing algorithms.

## 1. Introduction

With the development of wireless communication, sensor integration, and MEMS (micro- electro-mechanical systems), as well as progress in networking technology, wireless sensor network (WSN) technology and its industrial applications have been greatly improved and expanded, and the industrial WSN is attracting increasing attention [1,2]. Industrial WSN is a kind of ad hoc and cooperative network composed of a large number of industrial sensors and actuator nodes with wireless communication functions in industrial monitoring and control sites [3]. Integrated with various pressure, flow and temperature sensors and actuators, industrial WSNs are widely used in industrial field information collection and the feedback control of actuators, such as monitoring in petrochemical processes, offshore oil exploitation, long-distance transmission of oil and gas, and hazardous chemical production areas. Compared with conventional WSN, industrial WSN has higher requirements in system invulnerability, network connectivity and real-time performance [4,5]. However, the harsh industrial environment (e.g., high temperature and humidity, strong vibration, strong electromagnetic interference, etc.) often leads to wireless signal attenuation or interruption, and causes various defects during transmission, such as dispersion, delay, interference, and safety-related problems [6,7]. Accordingly, this will result in data error or packet loss, so that the on-site situation cannot be reflected to the factory control room in time, resulting in serious production accidents. In addition, these industrial sensor nodes are usually deployed remotely and are battery-driven in the unattended states, and nodes are vulnerable to malicious attacks or energy exhaustion [8,9], which leads to network partitioning, or even paralyzes the entire network. Therefore, how to improve the invulnerability of industrial WSNs is a research hotspot [10].

Real-world networks, such as the Internet, power grids, biological networks, and ad hoc networks, play an important role in modern society. All of these real networks in network science can be represented by complex networks. Increasing attention has been paid to the robustness of complex networks under connections or node failures. There are three well-known types of complex network models: random network models, like the Erdös–Rényi (ER) network model [11], small-world network models [12], and scale-free network models [13]. Albert proved that scale-free networks are highly resistant to random attack on nodes or edges, and vulnerable to deliberate attacks on nodes with a large number of connections [14]. The degree distribution of a scale-free network approximately follows the “power-law distribution”, therefore, the nodes with small node degree account for the majority in the network, and the nodes with large node degree account for the minority. However, the failure of nodes with low node degree has no obvious effect on the connectivity of the network. Obviously, constructing a scale-free industrial WSN topology can make it more invulnerable [15].

In short, the invulnerability of the network refers to the ability of the network to keep working after being subjected to deliberate or random attacks. Network invulnerability is divided into static invulnerability and dynamic invulnerability, and dynamic invulnerability is also called cascade invulnerability. The main difference between the two is that the failure of one node or link can lead to the failure of other nodes or links in the case of dynamic invulnerability, while the failure of one node or link will not affect the failure of other nodes or links in the case of static invulnerability. If a sensor node fails in an industrial WSN and the load is redistributed to adjacent nodes, the increasing load may cause cascading problems in the whole network, which will seriously affect the performance of the network and even lead to the collapse of the whole network. Therefore, when designing the topology of an industrial WSN, not only we should consider the network performance, but we should also consider the cascade failure. At present, the research on invulnerability is mainly based on cascade invulnerability. Motter et al. [16] proposed a simplified model of cascading failure and analyzed the causes of cascading failure. Souza et al. [17] further found that the cascade failure of a scale-free network has power-law characteristics. Dobson et al. [18] proposed a cascading failure control optimization algorithm based on node importance. None of the above studies considered the impact of weights of data traffic between two nodes, so Cui et al. [19] proposed an edge-weighting model related to node degree and intermediate centrality in order to control the spread of cascade failures in complex networks. In industrial WSNs, the topology change of the network will cause the data flow in the network to be redistributed, which will cause dynamic change of the network load. Therefore, a cascading failure model based on load redistribution is proposed in [20]. Since the nodes and links of the real network have the function of processing and transmitting data, the failure of one link or some of the nodes causes the load to be redistributed, which leads to the failure of the other nodes, but further load redistribution will lead to cascading failure, so Wang et al. [21] studied the impact of load redistribution on invulnerability. Ren et al. [22] proposed another cascade failure model based on residual energy of nodes. The model takes into account the residual energy of nodes during load redistribution, which makes full use of the network resources and improves the invulnerability of the network. Yin et al. [23] proposed a cascade failure model based on variable load and fixed storage capacity of the nodes, and solved the critical value of the load in the cascading failure. Zheng et al. [24] proposed a network topology reconstruction method based on adding edges with limited resources. Hu et al. [25] presented a regular hexagonal-based clustering scheme (RHCS) and a scale-free topology evolution mechanism (SFTEM) for WSNs, which increased network invulnerability as well as maintaining the energy balance. Qiu et al. [26] proposed a robustness optimization scheme with multi-population co-evolution for scale-free wireless sensor networks (ROCKS) to improve the robustness of the scale-free topology. Fu et al. [27] built a cascading model of clustering WSNs by introducing the concept of sensing load and relay load, and discussed the impacts of model parameters on network invulnerability. However, the above research almost all relies on optimizing the nodes performance to control cascading failures. Some of them only focus on the single node or edge which are attacked and unable to communicate, and some of them only analyze the static connection performance of the network, and do not consider how to construct the topology with strong connectivity and strong invulnerability under the cascading failure of the network. Our aim is to take into account the dynamic characteristics of the network, and construct a network topology with the strongest connectivity by an intelligent optimization algorithm, then study the invulnerability of the optimized network under the condition of cascade failures.

This paper builds an industrial WSN with scale-free characteristics, and proposes a topology optimization algorithm: fireworks and particle swarm optimization (FW-PSO). By making appropriate changes to model variables and constraints, the FW-PSO algorithm was applied to this kind of network to find the network topology with the maximum natural connectivity, and the dynamic and static invulnerability of the optimized network were analyzed under different attack strategies. The structure of the article is arranged as follows. The second part mainly introduces the construction of a scale-free network and cascade failure model. The third part mainly introduces the idea of the FW-PSO algorithm and the specific process of optimizing the scale-free WSN topology based on the FW-PSO algorithm. The fourth part is the experimental simulation and invulnerability analysis before and after network topology optimization. The last part concludes the research of this paper.

## 2. Scale-Free Network and the Cascading Failure Model

### 2.1. Scale-Free Network

In order to construct an industrial WSN model with scale-free characteristics, it is necessary to investigate the scale-free network construction process [28]:

(1) Growing

Suppose a network starts with a small number of $m_{0}$ nodes and $l_{0}$ edges. In each iteration, if a new node is added, $m\left(m\leq m_{0}\right)$ edges will be generated to connect to the existing nodes in the network at the same time.

(2) Preferential connection

When a new node joins the network, it will select the existing node to connect. Assuming that a new node is connected to other node *i* with a probability of $\Pi \left(k_{i}\right)$, the probability of the connection depends on the degree of node *i*, and its probability is expressed by:

(1) $$\Pi \left(k_{i}\right)=\frac{k_{i}}{\sum_{j}k_{j}}$$

Because the scale-free network is connected preferentially when it is formed, it exhibits the characteristics of power-law distribution, which makes the scale-free networks more fault-tolerant, but it shows strong vulnerability when it is deliberately attacked.

In order to better represent and understand the scale-free networks, many scholars have proposed that the measurement method represents the structural characteristics of the scale-free network. Here are some main feature metrics of scale-free networks:

(1) Degree of network nodes

In a network, the degree of a node refers to the number of nodes adjacent to the node, that is, the number of edges connecting the nodes. The degree of the network (*K*) refers to the average value of the degree of all the nodes in the network. The degree distribution P(k) refers to the probability of selecting any node in the network, whose degree is exactly *k*.

(2) Network aggregation coefficient 

If there are $k_{i}$ nodes around node *i*, the number of interconnections between the nodes is $E_{i}$, and the ratio of the number of these edges to the number of edges that may exist in this $k_{i}$ node is called the aggregation coefficient, which is represented by $C_{i}$ as Formula (2).

(2) $$C_{i}=\frac{2E_{i}}{k_{i}\left(k_{i}-1\right)}$$

The aggregation coefficient indicates the degree of connectivity between the nodes, which is the manifestation of network localization characteristics. The average aggregation coefficient refers to the average aggregation coefficient of all the nodes.

(3) Network average shortest path length

The shortest path $l_{ij}$ from node *i* to node *j* refers to the path with the least number of nodes in all the connected paths from node *i* to node *j*. The average shortest path L is an average for all $l_{ij}$. It is defined as Formula (3).

(3) $$L=\frac{1}{N\left(N-1\right)}\sum_{i\neq j}l_{ij}$$

Obviously, the above formula measures the connectivity and efficiency of the network, but it is no longer applicable when the network is disconnected, because L=∞ exists while it is disconnected. In order to better describe the connectivity of the network, the concept of overall network efficiency is proposed, which is defined as Formula (4).

(4) $$E=\frac{1}{N\left(N-1\right)}\sum_{i\neq j}\frac{1}{l_{ij}}$$

The above formula can measure the connectivity of the network under any circumstances. This index is used to measure the overall connectivity of the optimized network while it is attacked.

### 2.2. Establishment of Cascading Failure Model

In a scale-free network, when a node fails, the unprocessed data will be reassigned to its neighbors. In order to maintain network traffic and avoid network congestion, high-capacity neighbor nodes will be reallocated more unprocessed data [29]. Therefore, in this paper, the cascaded failure model based on the load priority redistribution principle is proposed, in which the initial load of nodes is set as a function of node degree.

(1) The relationship of the initial load $L_{j}$ of each node *j* in the network and its degree $k_{j}$ is defined as Formula (5).

(5) $$L_{j}=\beta k_{j}^{\alpha }$$

where, α and β control the strength of the initial load of the node, which are all adjustable parameters. Similar load assignment methods are used in many cascade failure models, such as WSNs, industrial Internet, power communication networks and so on [30].

(2) The load of node *i* is reassigned to neighbor node *j* according to the priority principle. The principle of node load redistribution is described as:

(6) $$\underset{j}{\Pi }=\frac{\beta k_{j}^{\alpha }}{\sum_{n\notin \Gamma_{i}}\beta k_{n}^{\alpha }}$$

where, $\Gamma_{i}$ is the set of all neighbor nodes on node *i*.

According to the principle of load redistribution, the additional load $\Delta L_{ij}$ received by node *j* from node *i* after node *i* fails is as follows

(7) $$\Delta L_{ij}=L_{ij}\frac{k_{j}^{\alpha }}{\sum_{n\in \Gamma_{i}}k_{n}^{\alpha }}$$

It can be seen from the above equation that the additional load $\Delta L_{ij}$ received by node *j* is independent of the selection of the parameters β, so the setting of β does not play a role in this additional load.

**Figure 1:**
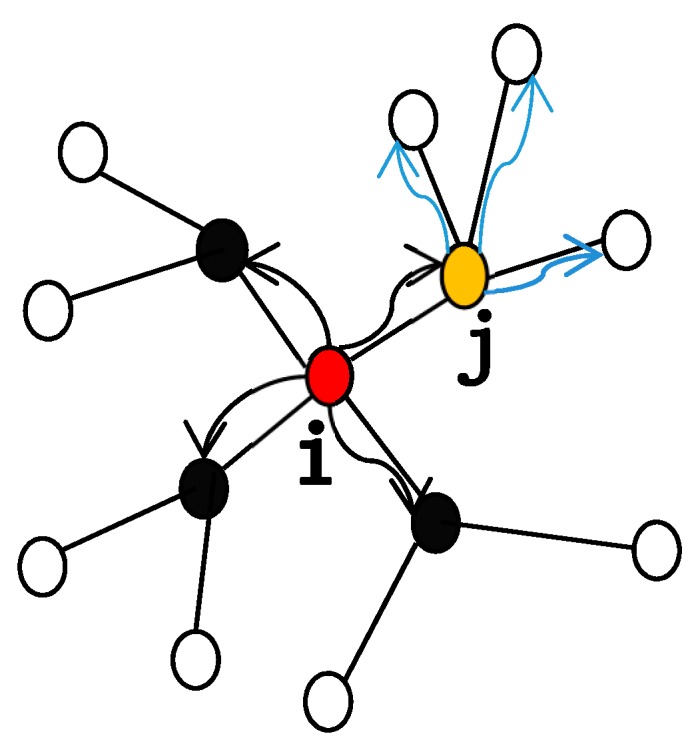
Re-localizing preferential allocation after a node fails.

(3) Node capacity reflects the load-carrying capacity and is constrained by network cost. Assuming that the capacity $Ca_{j}$ of node *j* is proportional to its initial load, then $Ca_{j}$ is described as:

(8) $$Ca_{j}=TL_{j},\;j=1,2,3,\ldots ,N$$

where, the value of T is greater than or equal to 1. If node *j* receives an extra load that exceeds its capacity, that is, $L_{j}+\Delta L_{ji}>C_{j}$, node *j* will fail. The failure of node *j* leads to further load redistribution, which may trigger the failure of other nodes and cause the cascading failures. The following Figure 1 shows the process of re-localizing preferential allocation after a node fails. As shown by the black line arrow, the red node *i* in the center fails, which causes its load to be distributed to the neighbors: the black nodes and *j*-node. However, the *j*-node receives the extra load beyond its capacity, which leads to its own failure, so the *j*-node begins to distribute the load to its neighbor nodes, as shown by the light blue line arrow in the figure, resulting in cascading failure of the network. 

In this paper, $CF_{i}$ is used to indicate the number of failed nodes caused by node *i* [31]. Obviously, it holds $0\leq CF_{i}\leq N-1$. In order to quantify the global cascade features of the whole network caused by attacks on some nodes, the normalization index of some nodes after being attacked is introduced, that is:

(9) $$CF_{attack}=\frac{\sum_{i\in A}CF_{i}}{N_{A}\left(N-1\right)}$$

where, *A* represents the set of attacked nodes and $N_{A}$ represents the number of attacked nodes.

Through the analysis of the above cascaded fault model, we can see that the size of the node fault tolerance parameter T directly affects whether the network is faulty or not. When T is large, the failure of any node cannot cause cascading failure. When T is very small, the failure of any node will lead to network paralysis. Therefore, the network has a phase transition from the free phase to the congestion phase, which is the critical threshold $T_{c}$. When $T\geq T_{c}$, each node in the network can handle the additional load from other nodes without cascading failure, and the network can run normally. However, when $T<T_{c}$, because each node has a limited ability to deal with failures, $CF_{attack}$ suddenly grows rapidly from 0, causing the entire network or part of the network to fail.

It is obvious that $T_{c}$ is the minimum value of the fault-tolerance ability of nodes used to avoid cascading faults. The smaller the value of $T_{c}$ is, the stronger the invulnerability of the network in the case of cascading failure will be, so the critical threshold $T_{c}$ can well reflect the invulnerability of the network. In this paper, the size of $T_{c}$ is used to measure the invulnerability to resist attacks after network optimization. 

## 3. The Process of Optimizing the Scale-Free Network Based on the Fireworks and Particle Swarm Optimization (FW-PSO) Algorithm

### 3.1. Overview of PSO and Fireworks Algorithms

(1) PSO Algorithm

In the PSO algorithm, if the total number of particle swarms is *n*, the search space is *D* dimension, the position of the *i*-th particle is represented as $x_{i}=\left(x_{i1},x_{i2},x_{i3},\ldots ,x_{iD}\right)$, and the optimal position of the *i*-th particle currently searched is $pbest_{i}=(P_{i1},P_{i2},\ldots ,P_{iD})$, and the current optimal position of the entire particle swarm searched is $gbest=\left(g_{1},g_{2},g_{3},\ldots ,g_{D}\right)$, the velocity change rate of the *i*-th particle is $v_{i}=\left(v_{i1},v_{i2},\ldots ,v_{iD}\right)$, and the velocity and position of each iteration of a single particle are expressed as follows [32]:

(10) $$v_{id}(t+1)=w\cdot v_{id}(t)+c_{1}\cdot r_{1}\cdot \left(p_{id}(t)-x_{id}(t)\right)+c_{2}\cdot r_{2}\cdot \left(p_{gd}(t)-x_{id}(t)\right)$$

(11) $$\begin{array}{c} x_{id}(t+1)=x_{id}(t)+v_{id}(t+1),\\ 1\leq i\leq n,1\leq d\leq D \end{array}$$

where, $c_{1}$ and $c_{2}$ are the acceleration factors, and are all the constant. $r_{1}$ and $r_{2}$ are the random numbers between [0, 1]. w is the inertia weight, and w plays the role of balancing the global search and the local search. When the inertia weight w is large, the global search ability is strong and the local search ability is weak [33]. Instead, when the inertia weight w is small, the local search ability is enhanced, and the global search ability is weakened. A large number of experimental results show that the convergence rate of particle swarm is faster when the inertia weight is between [0.8, 1.2]. Most of the adjustment of w is adopted as the linear decreasing weight strategy (LDW) [34], which is defined as:

(12) $$w=w_{\max}-\left(w_{\max}-w_{\min}\right)\cdot \frac{iter}{iter_{\max}}$$

where, $w_{\min}$ is the minimum inertia weight, $w_{\max}$ is the maximum inertia weight, *iter* is the current iteration number, and $iter_{\max}$ is the total number of iterations of the algorithm.

(2) Fireworks Algorithm

The fireworks algorithm obtains the optimal solution through continuous iteration, which mainly consists of three parts: the explosion operator, the Gaussian mutation operator and the selection strategy. In the fireworks algorithm, the fireworks represent the potential feasible solution of the optimization problem, and the process of fireworks generating sparks represents the search in the feasible solution space. In each iteration, sparks are generated in two ways: explosion and Gaussian variation. The explosion of fireworks is mainly controlled by the explosion radius and the number of explosion sparks [35]. Assuming that the number of fireworks is *Num*, the explosion radius $A_{i}$ of the *i*-th fireworks $x_{i}$ (i=1,2,…,Num) and the number of explosion sparks $S_{i}$ are calculated by Formulas (13) and (14):

(13) $$A_{i}=A\times \frac{f\left(x_{i}\right)-y_{\min}+\varepsilon }{\sum_{i=1}^{N}\left(f\left(x_{i}\right)-y_{\min}\right)+\varepsilon }$$

(14) $$S_{i}=M\times \frac{y_{\max}-f\left(x_{i}\right)+\varepsilon }{\sum_{i=1}^{N}\left(y_{\max}-f\left(x_{i}\right)\right)+\varepsilon }$$

where, *A* and *M* are the constants, which are used to adjust the explosion radius of fireworks and the number of explosion sparks generated. $f\left(x_{i}\right)$ represents the fitness value of fireworks $x_{i}$, $y_{\min}=\min\left(f\left(x_{i}\right)\right)$, $y_{\max}=\max\left(f\left(x_{i}\right)\right)$. ε is the machine precision, which is used to avoid dividing by zero. The boundary of $S_{i}$ is defined as:

(15) $$S_{i}=\left\{ \begin{array}{ll} round\left(a*M\right), & S_{i}<a*M\\ round\left(b*M\right), & S_{i}>b*M\\ round\left(S_{i}\right), & othersize \end{array}\right.$$

where, *a* and *b* are the limiting factors of explosion number, both of which are the constants.

In order to increase the diversity of explosion fireworks, the Gaussian variation operation is introduced. The way fireworks $x_{i}$ performs Gaussian mutation operation on dimension *k* is:

(16) $${\hat{x}}_{ik}=x_{ik}\times e$$

where *e* is a Gaussian distribution with the mean and variance of 1.

In order to transmit the information to the next generation, a new fireworks population will be selected for iteration. After the explosion spark and Gaussian variation spark are generated through the above steps, a certain number of individuals from all fireworks, explosion spark and Gaussian variation spark will be selected as the next generation of fireworks for iteration. Among them, the one with the best fitness is selected into the next generation with certainly, and the remaining *Num-1* fireworks are selected by roulette. The selection operation is as follows:

(17) $$P\left(X_{i}\right)=\frac{R\left(X_{i}\right)}{\sum_{k\in K}R\left(X_{k}\right)}$$

(18) $$R\left(X_{i}\right)=\sum_{j\in K}d\left(X_{i},X_{j}\right)=\sum_{j\in K}\left\|X_{i}-X_{j}\right\|$$

where, *K* represents the set of all fireworks and the two sparks, $R\left(X_{i}\right)$ represents the sum of the distances between the current individual and the remaining other individuals. $P\left(X_{i}\right)$ indicates the probability that the current fireworks are selected.

(3) FW-PSO Algorithm

For the PSO algorithm, the particle can quickly find a better solution under the guidance of its historical optimal solution and the current global optimal solution, and the convergence speed is fast. However, since the update of the particle position in the particle swarm is mainly evolved by comparing its own position, the surrounding position and the current optimal position in particle swarm, it lacks global comparison measures. Therefore, the convergence speed is not high in the later calculations, and it is easy to fall into the local optimum. In the fireworks algorithm, fireworks can find global optimal solutions in the entire search space through explosion and mutation operations. In order to take advantage of the two algorithms, this paper proposes a FW-PSO algorithm.

**Figure 2:**
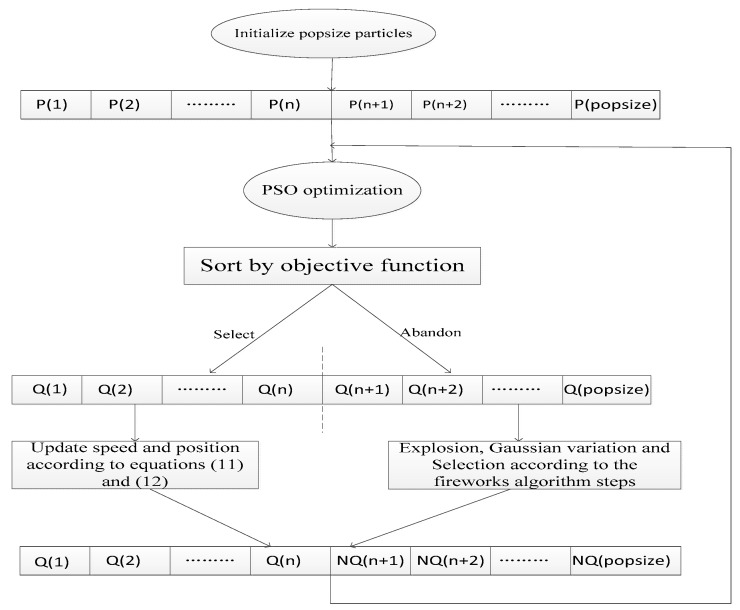
The schematic diagram of the fireworks and particle swarm optimization (FW-PSO) algorithm.

After the FW-PSO algorithm first evolves a certain number of iterations by PSO algorithm, it selects *n* particles with the best fitness and keeps them, and deletes *popsize-n* particles with poor fitness at the same time, where *popsize* is the population size. Then, the remaining *n* particles are subjected to explosion, mutation and selection operations to obtain *popsize-n* particles. Finally, the *n* particles retained by PSO and the *popsize-n* particles obtained by fireworks algorithm are combined to form a new particle swarm to continue the next iteration. The schematic diagram is shown as Figure 2.

### 3.2. Scale-Free Topology Optimization Model of Industrial Wireless Sensor Network (WSN) and Optimization Solution Process

(1) Scale-Free Topology Optimization Model of Industrial WSN

The invulnerability of the network refers to the ability of the network to continue to work after being attacked. This ability to continue working is mainly reflected in the ability of the network to maintain connectivity. It is shown by [36] that the natural connectivity is strictly monotonous, which can reflect the invulnerability performance of the network well. Therefore, this paper takes natural connectivity as the objective of invulnerability optimization to find the topology with the maximum connectivity and analyzes the invulnerability of the optimized network in the case of failure.

Industrial WSN can be described by an unweighted and undirected graph G=(V,E), where $V=\left\{v_{1},v_{2,\ldots ,}v_{N}\right\}$ is a group of nodes and $E=\left\{e\left(v_{i},v_{j}\right)\right\}$ is a group of edges; where N=|V| is the total number of nodes in the network and W=|E| is the number of edges. In order to model the networks, the following definitions are given.

**Definition** **1.** *(Adjacency matrix):*$A\left(G\right)={\left(a_{ij}\right)}_{N\times N}$*is defined as the adjacency matrix of a graph, then the graph G can be represented by its adjacency matrix; where, the value set of*$a_{ij}$*is*$\left\{a_{ij}=a_{ji}=1|e\left(v_{i},v_{j}\right)\in E\left(G\right)\right\}$*or*$\left\{a_{ij}=a_{ji}=0|e\left(v_{i},v_{j}\right)\notin E\left(G\right)\right\}$.

**Definition** **2.** *(Laplace matrix): If* $L\left(G\right)\in R\times R^{N}$ *is a Laplacian matrix of graph* G *, then* L(G) *is defined as* $L\left(G\right)=\overset{\wedge }{D}\left(G\right)-A\left(G\right)$ *, where* $\overset{\wedge }{D}\left(G\right)=diag\left\{d_{i}\right\}$ *is the diagonal matrix formed by the degrees of the nodes.*

According to graph theory, the Laplace matrix of graph G shows some remarkable properties in its connectivity. This is assuming the eigenvalues of the Laplacian matrix L(G) are $\mu_{i}$, i=1,2,…,N. Sorting the eigenvalues from large to small gives: $\mu_{N}\geq \mu_{N-1}\geq \ldots \geq \mu_{2}\geq \mu_{1}=0$.

**Definition** **3.** *(Algebraic connectivity): Define* $\mu =\mu_{2}$ *, if and only if* μ>0 *, the graph is connected.* μ *is the algebraic connectivity of the graph.*

It can be seen from [37] that the redundancy of the alternative path in a network is closely related to the eigenvalue of its adjacency matrix. If there are multiple paths between two nodes $v_{i}$ and $v_{j}$, when one of the paths fails, the two nodes can still communicate through other paths. That is, the more redundant paths between the nodes are, the more invulnerable the network is. Therefore, the redundancy of alternative paths in the network can reflect the invulnerability of the network. In order to measure the redundant paths in the network, it is generally necessary to count the number $n_{ij}^{l}$ of the paths with the length l between any pair of nodes $v_{i}$ and $v_{i}$, and then sum them as:

(19) $$R=\sum_{i=1}^{N}\sum_{j=1}^{N}\sum_{l=0}^{\infty }n_{ij}^{l}$$

However, $n_{ij}^{l}$ is difficult to calculate, and *R* would be a rather complex expression. Therefore, the number of closed paths in the network is considered to measure the redundant paths in the network. The above equation can be written as:

(20) $$R=\sum_{i=1}^{N}\sum_{l=0}^{\infty }n_{i}^{l}=\sum_{l=0}^{\infty }\sum_{i=1}^{N}n_{i}^{k}=\sum_{l=0}^{\infty }n_{l}$$

where, $n_{i}^{l}$ is the number of closed pathways where both the start and end points are $v_{i}$ and the length is *l*. $n_{l}$ represents the number of closed paths of length *l* in the network. Since the paths in the network allow nodes and edges to repeat, the length of the closed path can be arbitrarily long, namely R→∞. It is also considered that the shorter closed paths have a greater impact on the redundancy of alternative paths, so we can divide *R* by the factorial of length *l* to measure the contribution of closed paths. Then *R* can be revised and expressed as:

(21) $$R=\sum_{l=0}^{\infty }\frac{n_{l}}{l!}$$

**Corollary** **1.** *If R is the sum of path number*$n_{ij}^{l}$*with length l between any node pair*$v_{i}$*and*$v_{j}$*in the network, it can be simplified as*$R=\sum_{i=1}^{N}e^{\lambda_{i}}$.

**Proof.**  Referring to Formula (20), it holds that $R=\sum_{l=0}^{\infty }\frac{n_{l}}{l!}$. To simplify this formula, the following lemma is given.

**Lemma** **1.** *$n_{l}$ represents the number of closed paths with length l in the network, then,*

(22) $$n_{l}=trace\left(A^{l}\right)=\sum_{i=1}^{N}\lambda_{i}^{l}$$

*By using the expression of $n_{l}$, *R* can be simplified as:*

(23) $$\begin{array}{l} R=\sum_{l=0}^{\infty }\frac{n_{l}}{l!}=\sum_{l=0}^{\infty }\sum_{i=1}^{N}\frac{\lambda_{i}^{l}}{l!}\\ =\sum_{i=1}^{N}\sum_{l=0}^{\infty }\frac{\lambda_{i}^{l}}{l!}=\sum_{i=1}^{N}e^{\lambda_{i}} \end{array}$$

□

It can be seen from the above equation that the number of closed paths in the network can be obtained according to the eigenvalues of its adjacency matrix. When *N* is very large, *S* is also very large, so *R* can be transformed as:

(24) $$\bar{\lambda }==\ln\left(\frac{R}{N}\right)=\ln\left(\frac{1}{N}\sum_{i=1}^{N}e^{\lambda_{i}}\right)$$

As seen from the expression above, $\bar{\lambda }$ corresponds to the ‘average eigenvalue’ of the graph adjacency matrix.

**Definition** **4.** *(Natural connectivity): assuming the eigenvalue of the adjacency matrix* A(G) *is* $\lambda_{i}$ *, then the natural connectivity of graph* G *is:*

(25) $$\bar{\lambda }=\ln\left(\frac{1}{N}\sum_{i=1}^{N}e^{\lambda_{i}}\right)$$

According to the above analysis, natural connectivity describes the redundancy of alternative paths in the network from the internal structure of the network. The larger the natural connectivity, the better the invulnerability of the network will be. Therefore, finding the network topology with the greatest natural connectivity is of great significance to the improvement of network invulnerability [38]. Based on the above analysis, it is feasible and reasonable to use the maximum natural connectivity as the optimization goal. The natural connectivity is strictly and monotonically increasing for the added edges, which means that the natural connectivity can accurately reflect the slight difference in invulnerability. However, the network is limited by the cost, the number of network edges is bound to be limited, so the constraint condition of edge is set as: $W=\left|E\right|=\frac{1}{2}\sum_{i=1}^{N}\sum_{j=1}^{N}a_{ij}$. In the process of topology optimization, the connectivity of the graph needs to be ensured. Considering the accuracy problem in the calculation process, the algebraic connectivity is set to μ>0.01. Otherwise, isolated nodes will appear in the topology graph to make the network disconnect. According to the above analysis, the topological structure of the network is optimized, and the optimization function is set as:

(26) $$\max\bar{\lambda }=\ln\left(\frac{1}{N}\sum_{i=1}^{N}e^{\lambda_{i}}\right)$$

The constraints are as follows:

(27) $$\begin{array}{l} \sum_{i=1}^{N}\sum_{j=1}^{N}a_{ij}=2W\\ \mu >0.01 \end{array}$$

(2) The Solution Process of FW-PSO Algorithm

Since FW-PSO algorithm is suitable for solving the continuous optimization problems, a variable transformation is required for the topology optimization model. The specific operations are as follows:

(i) In the Formula (26), $a_{ij}$ represents the lower triangular matrix of the adjacency matrix A(G) (excluding the diagonal elements), that is, i>j. Therefore, the N(N−1)/2 elements are rearranged and recorded into $X=\left(x_{1,}x_{2},\ldots ,x_{N\left(N-1\right)/2}\right)$.

(ii) Convert the variable to a continuous variable. In the X obtained in the previous step, $x_{i}$ is 0 or 1. If $X^{\prime }=g\left(X\right)$ is ordered, the g(X) is the mapping from X to $X^{\prime }$, and thus $x_{i}^{\prime }=\left\{ \begin{array}{ll} 0, & x_{i}<0.5\\ 1, & x_{i}\geq 0.5 \end{array}\right.$.

(iii) In order to ensure that the constraint $\sum_{i=1}^{N\left(N-1\right)/2}x_{i}^{\prime }=W$ of the edge is still satisfied after the mapping, that is, the number *M* of $x_{i}\geq 0.5$ is *W* in *X*. In the process of optimizing the particle swarm, the number *M* of $x_{i}\geq 0.5$ may appear to be greater or less than W. In view of this, the variable is adjusted as follows: 

In the case M<W, we randomly extract *W*-*M* numbers of $x_{i}<0.5$ in X, and replace them with the numbers greater than 0.5 randomly generated between (0, 1).

In the case of M>W, we randomly extract *M*-*W* numbers of $x_{i}\geq 0.5$ in X, and replace them with the numbers less than 0.5 randomly generated between (0, 1). 

According to the above analysis, the steps for optimizing the network topology based on FW-PSO are as follows:

**Step1**: According to the construction algorithm of scale-free network, the industrial WSN with scale-free characteristics is generated and its adjacency matrix A(G) is obtained.

**Step2**: The elements in the adjacency matrix A(G) are transformed according to (1), (2) and (3) above. 

**Step3**: Initialize the parameters of FW-PSO, including the weight coefficient: $w_{\min}$ and $w_{\max}$, the population size: *popsize*, the acceleration factor: $c_{1}$ and $c_{2}$, the explosion radius adjustment factor *A*, and the explosion number adjustment factor: *M*, *a* and *b*. The number of particles retained by PSO optimization: *n*, the number of iterations of PSO: *maxgen*, and the number of iterations of FW-PSO: $gen_{\max}$.

**Step4**: The optimization function is solved according to the principle of FW-PSO algorithm in Section 3.2.

**Step5**: Output the optimal fitness value and the corresponding position obtained by Step4.

The specific pseudo code for the optimization solution using the FW-PSO algorithm is as Algorithm 1.
**Algorithm 1.** The pseudo code of the solving process of the FW-PSO algorithm-- *fpbest*: The best fitness value of the individual-- *fgbest*: The best fitness value of the group**Input:** Objective function *f(x)* and the constraint conditionParameters initialization: including *popsize, n*, $c_{1}$, $c_{2}$, $w_{\max}$, $w_{\min}$ Total iterations $gen_{\max}$, PSO iterations *maxgen*, *A, M, a* *and b*Group initialization: random initialization position $x_{i}$ and velocity $v_{i}$ of particles, calculate fitness *fgbest* of particles using Equation (18)Set $fpbest_{i}\leftarrow x_{i}(x_{i}\in \left[1,2,3,\ldots ,popsize\right])$, $gen_{\max}\leftarrow 1$**While***gen* < $gen_{\max}$   **for** *pgen*←1 to *maxgen*     **for** i←1 to *popsize*       Update $v_{i}$ and $x_{i}$ of particle by using (10) and (11)       Calculate $f\left(x_{i}\right)$       **if** $f\left(x_{i}\right)>fpbest\left(x_{i}\right)$         **then** $fpbest\left(x_{i}\right)\leftarrow f\left(x_{i}\right)$       **end if**       **if** $f\left(x_{i}\right)<fpbest\left(x_{i}\right)$         **then** $f\left(x_{i}\right)\leftarrow fgbest\left(x_{i}\right)$       **end if**     **end for**        pgen←pgen+1  **end for** Sort the particle group in descending order and select the *n* particles with better fitness. According to the position values of *n* particles, calculate the $A_{i}$ and $S_{i}$ by using (13) and (14). Generate mutation sparks by using (16). Select the *popsize-n* individuals from the fireworks, explosion sparks and mutation sparks by using the selection strategy (17). Combine the *n* particles with *popsize-n* individuals to generate the new population. Calculate *fpbest* and *fgbest* of the new group. gen←gen+1**end while****Output:***fgbest*

## 4. The Simulation Experiments and Analysis

### 4.1. Experimental Simulation of Optimizing Network Topology

In order to verify the effectiveness of the network topology optimization algorithm proposed in Section 3, MATLAB R2016a was used as experimental simulation platform. Suppose the node distribution area is 100 × 100 m^2^. The parameters setting are shown in Table 1.

**Figure 3:**
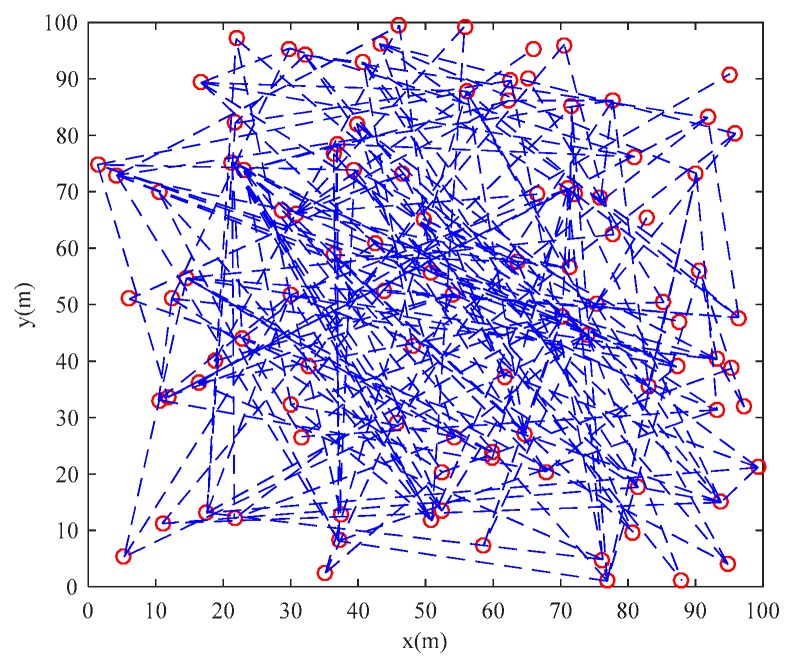
The initial topology of the network.

**Figure 4:**
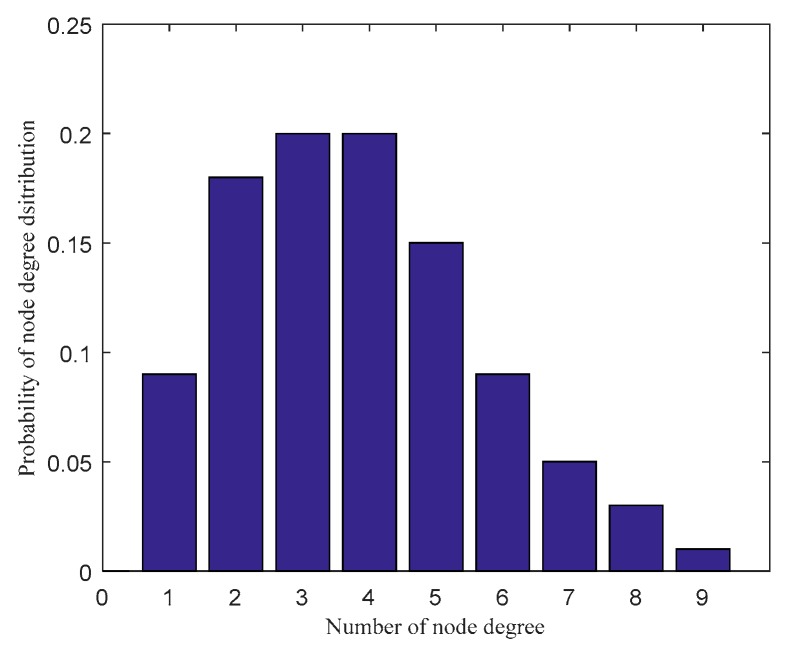
The nodes degree of distribution in the network before optimization.

**Figure 5:**
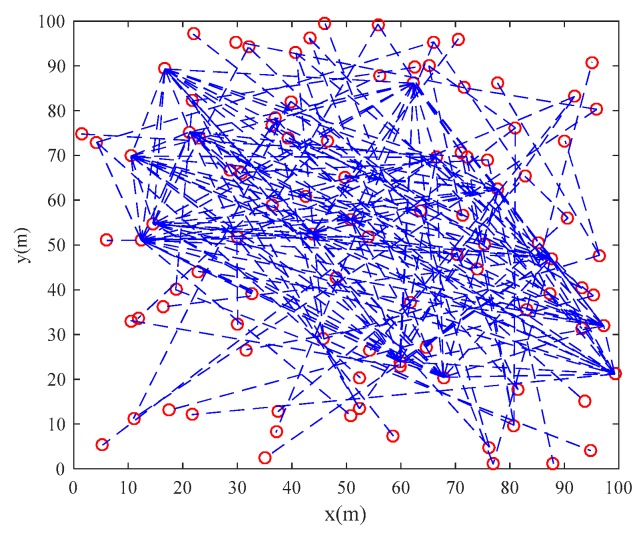
The optimized topology by FW-PSO.

**Figure 6:**
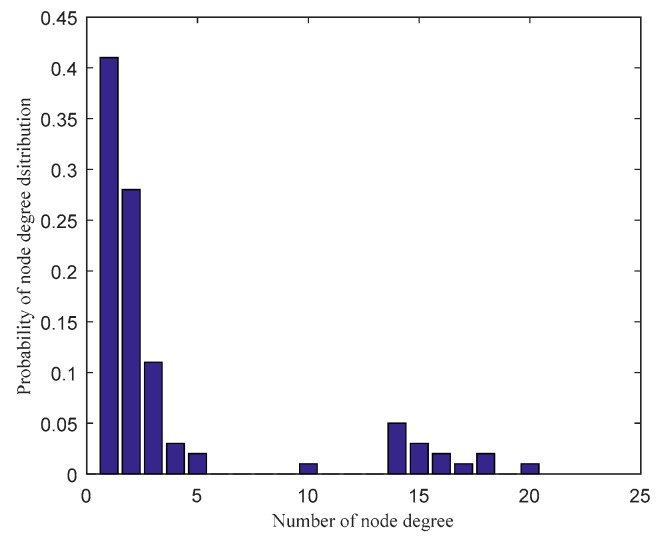
The nodes degree of distribution in the network after FW-PSO optimization.

Based on the parameters setting in Table 1 and the scale-free network construction algorithm in Section 2.1, the scale-free topology optimization model of industrial WSN and its degree of distribution diagram before optimization are generated as Figure 3 and Figure 4. According to the FW-PSO algorithm, the optimized industrial WSN topology and its degree of distribution are shown as Figure 5 and Figure 6. The connection in the topology represents the communication link between two nodes, and the degree of nodes represents the number of nodes’ connection links.

**Figure 7:**
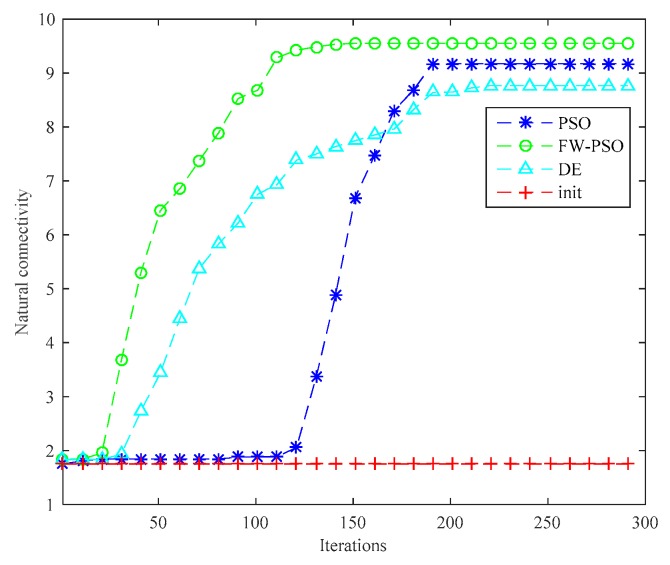
Relationship between natural connectivity and iterations with the comparison of different algorithms.

**Figure 8:**
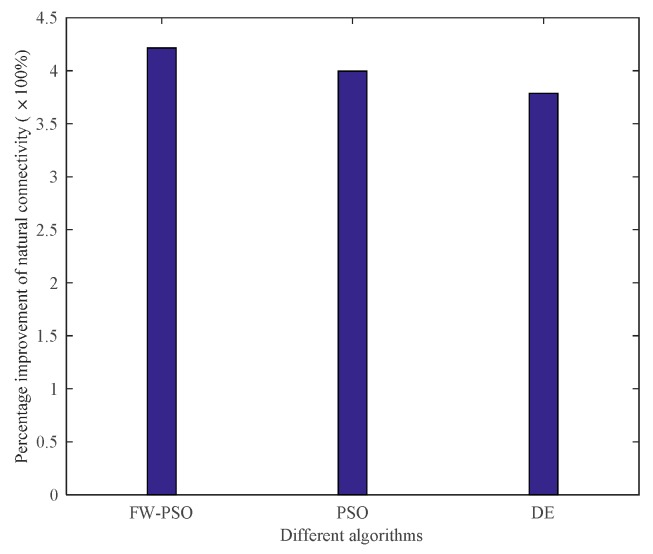
Percentage improvement in natural connectivity of different algorithms relative to the initial network.

From the topology and degree of distribution figures before and after the optimization, we can see that FW-PSO algorithm optimizes the network topology by changing the degree of nodes, the node position does not move, and the particles in the algorithm represent the position set of all the nodes. Figure 7 shows the relationship between the natural connectivity and the number of iterations. It can be seen from the figure that, with the increase of the number of iterations, the natural connectivity is increasing, which indicates that the invulnerability of the network is increasing. The final optimized natural connectivity of FW-PSO, the standard PSO and the differential evolution (DE) algorithms are 9.5526, 9.1523 and 8.7691, respectively. The natural connectivity of initial network topology (init) is 1.8322. The proposed FW-PSO has better optimizing results and faster convergence speed than PSO and DE algorithms. Figure 8 shows the percentage improvements of the natural connectivity optimized by FW-PSO, PSO and DE algorithms with respect to the initial natural connectivity. It can be seen from the figure that the proposed algorithm has the best improvement compared with the others.

### 4.2. Invulnerability Analysis

In order to study the invulnerability of network topology before and after optimization, this section will analyze the dynamic and static invulnerability. Firstly, the attack strategies are selected as HD (highest degree) and LD (lowest degree) to analyze the ability of the network to resist cascading failure before and after optimization, that is, the investigation of dynamic invulnerability. HD means attacking the nodes which have the high node degree in the network, and LD means attacking the nodes which have the low node degree in the network. Then, we would like to analyze the static invulnerability of the network under random attack.

(1) Dynamic Invulnerability

**Figure 9:**
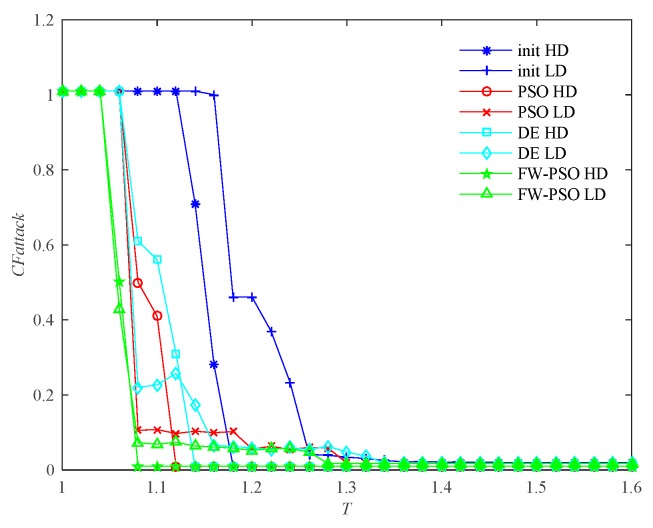
Comparison of two attack strategies before and after network optimization at α<1.

**Figure 10:**
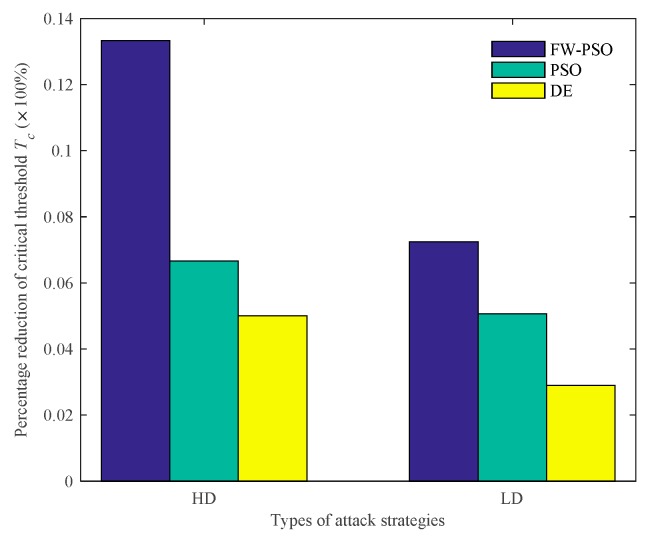
Percentage reduction of critical threshold *T_c_* after optimization of different algorithms under two attack strategies at α<1.

**Figure 11:**
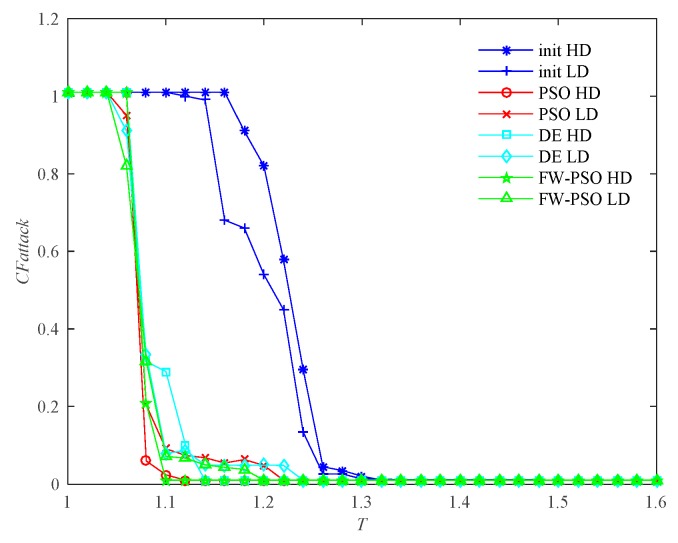
Comparison of two attack strategies before and after network optimization at α=1.

**Figure 12:**
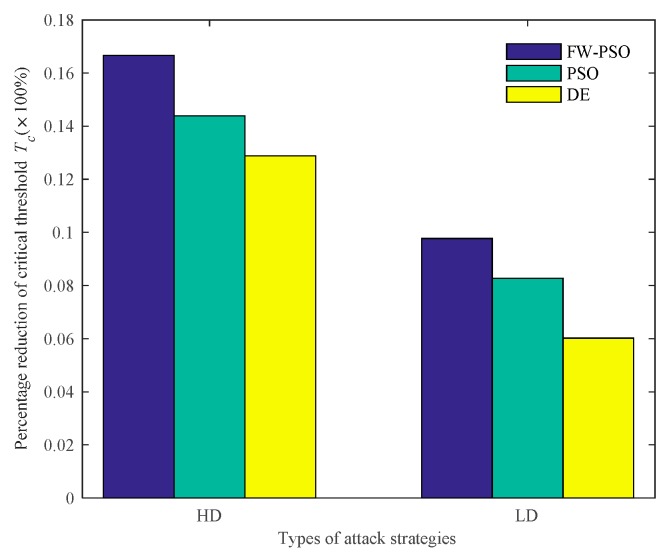
Percentage reduction of critical threshold *T_c_* after optimization of different algorithms under two attack strategies at α=1.

**Figure 13:**
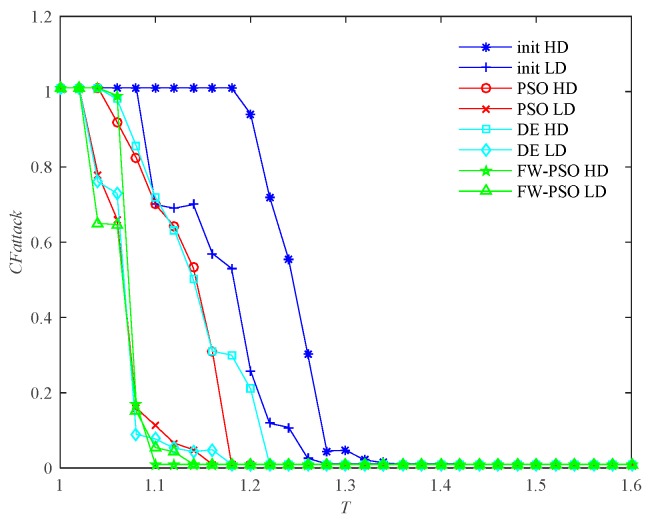
Comparison of two attack strategies before and after network optimization at α>1.

**Figure 14:**
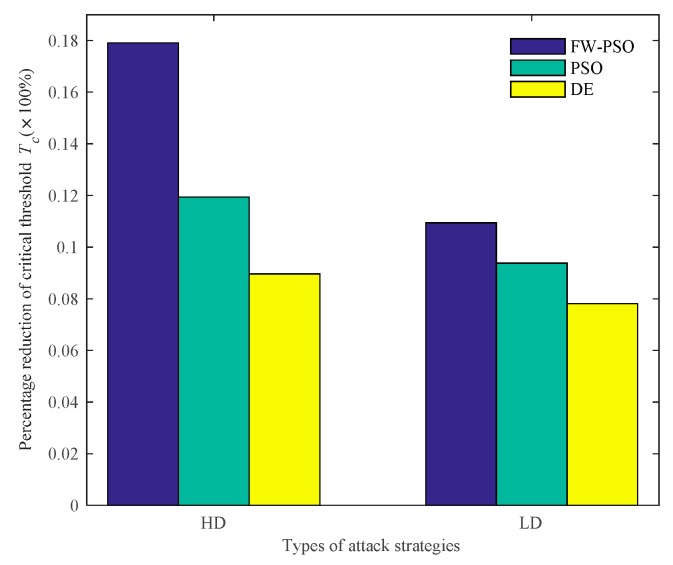
Percentage reduction of critical threshold *T_c_* after optimization of different algorithms under two attack strategies at α>1.

According to the cascading failure model mentioned in Section 2.2, we analyze the critical thresholds $T_{c}$ of the network topology before and after optimization by FW-PSO, standard PSO, and DE algorithms under the two kinds of attack strategies of HD and LD. In order to avoid contingency, 10 repeated experiments were conducted, and the average value was taken. Referring to [29], the invulnerability analysis was carried out while α is greater than, less than or equal to 1, and the percentage reductions of critical threshold $T_{c}$ after optimization of the three algorithms under two attack strategies were compared accordingly. These results are shown as Figure 9, Figure 10, Figure 11, Figure 12, Figure 13 and Figure 14, respectively.

In Figure 9, the abscissa *T* represents the node fault tolerance parameter, and the ordinate *CFattack* represents the normalization index to measure the global cascading fault features of the whole network when some nodes are attacked.

It can be seen from Figure 9 that, when α<1, the critical threshold $T_{c}$ of HD attack before the optimization is 1.20, the critical threshold $T_{c}$ after the optimization of DE, standard PSO and FW-PSO algorithms are 1.14, 1.12 and 1.08, respectively. The critical threshold $T_{c}$ of LD attacks before the optimization is 1.38, and the critical threshold $T_{c}$ after the optimization of DE, standard PSO, and FW-PSO algorithms are 1.34, 1.31 and 1.28, respectively. Figure 11 shows that, when α=1, the critical threshold $T_{c}$ of the HD attack before the optimization is 1.32, and the critical threshold $T_{c}$ after the optimization of DE, standard PSO and FW-PSO algorithms are 1.15, 1.13 and 1.10, respectively. The critical threshold $T_{c}$ of the LD attack before optimization is 1.33, and the critical threshold $T_{c}$ after the optimization of DE, standard PSO and FW-PSO algorithms are 1.25, 1.22 and 1.20, respectively. Figure 13 shows that, when α>1, the critical threshold $T_{c}$ of the HD attack before the optimization is 1.34, and the critical threshold $T_{c}$ after the optimization of DE, standard PSO and FW-PSO algorithms are 1.22, 1.18 and 1.10, respectively. The critical threshold $T_{c}$ of the LD attack before the optimization is 1.28, and the critical threshold $T_{c}$ after the optimization of DE, standard PSO and FW-PSO algorithms are 1.18, 1.16 and 1.14, respectively. As can be seen from Figure 10, when α<1, the percentage reductions of the critical threshold $T_{c}$ of the HD attack after the optimization of FW-PSO, standard PSO and DE algorithms are 13.33%, 6.67% and 5.00%, respectively. The percentage reductions of the critical threshold $T_{c}$ of the LD attack after the optimization of FW-PSO, standard PSO and DE algorithms are 7.24%, 5.07% and 2.90%, respectively. Figure 12 shows that, when α=1, the percentage reductions of the critical threshold $T_{c}$ of the HD attack after the optimization of FW-PSO, standard PSO and DE algorithms are 16.67%, 14.39% and 12.88%, respectively. The percentage reductions of the critical threshold $T_{c}$ of the LD attack after the optimization of FW-PSO, standard PSO and DE algorithms are 9.77%, 8.27% and 6.02%, respectively. Figure 14 shows that, when α>1, the percentage reductions of the critical threshold $T_{c}$ of the HD attack after the optimization of FW-PSO, standard PSO and DE algorithms are 17.91%,11.94% and 8.96%, respectively. The percentage reductions of the critical threshold $T_{c}$ of the LD attack after the optimization of FW-PSO, standard PSO and DE algorithms are 10.94%, 9.38% and 7.81%, respectively. As a result, it can be seen from Figure 9, Figure 10, Figure 11, Figure 12, Figure 13 and Figure 14 that the proposed algorithm reduces the critical threshold *T_c_* more than the other algorithms. Therefore, no matter what value α is taken, the network optimized by the proposed FW-PSO is significantly improved in the cases of HD or LD attacks, and the FW-PSO algorithm is better than DE and PSO algorithms in improving the network’s invulnerability.

(2) Static Invulnerability

**Figure 15:**
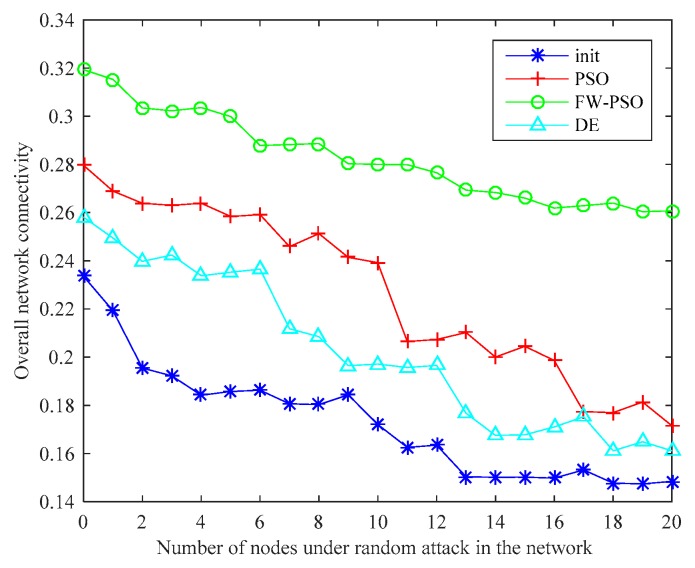
Network connectivity during random attacks.

**Figure 16:**
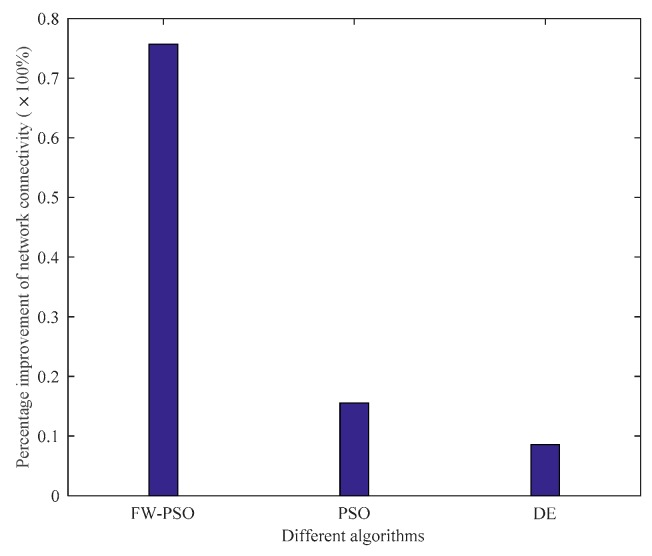
Percentage improvement in network connectivity of different algorithms.

The above analysis shows that, after the optimizations of DE, standard PSO and FW-PSO algorithms, the dynamic invulnerability of the network in the event of HD or LD attacks is improved effectively. In order to better verify the performance of the optimized network, the static invulnerability under random attacks also needs to be analyzed. The corresponding experimental results are shown as Figure 15 and Figure 16.

As seen in Figure 15, the connectivity of the optimized network under random attack is greatly improved compared with that before optimization, which shows that the invulnerability of the optimized network is significantly enhanced. Figure 16 shows the percentage improvement of the network connectivity with respect to the initial network connectivity after optimization by the FW-PSO, PSO, and DE algorithms, and it can be seen from the figure that the proposed algorithm has the best improvement effect. In addition, the network optimized by the FW-PSO algorithm has higher connectivity and stronger invulnerability in the face of random failures than that optimized by standard PSO and DE algorithms. Through the above analysis, it can be concluded that the network optimized by FW-PSO can also significantly improve survivability when subjected to random attacks. This indicates that the static invulnerability of the optimized network is also enhanced.

Due to the lack of effective oscillation and mutation measures, the PSO algorithm has a slow convergence speed in the later stage, and even falls into a local optimum. The DE algorithm has fixed crossover probability and crossover factor, which makes it easy to fall into premature problems. The proposed FW-PSO algorithm combines the advantages of the PSO algorithm’s fast optimization speed and the fireworks algorithm’s diversity of population, and it has obvious advantages in searching results and convergence speed. The scheme proposed in this paper combines the construction method of the scale-free network and the FW-PSO algorithm, so as to construct a scale-free industrial WSN with maximum natural connectivity, and solves the problem of network topology construction with strong invulnerability for industrial WSN. The proposed optimization method can effectively promote the ability of the network to resist the cascade failure problem, and the cascade failure model was explored to analyze the dynamic invulnerability in the case of some node failures, as well as the static invulnerability under random attack, rather than only focus on the single node or edge which are attacked and unable to communicate, or only analyze the static connection performance of the network as in previous work. The analysis results show that the proposed scheme makes the network have strong dynamic and static invulnerability, thus meeting the needs of industrial WSN for strong connectivity and invulnerability.

## 5. Conclusions

This paper proposes a FW-PSO algorithm, which combines the advantages of the population diversity of the firework algorithm and the strong ability of PSO searching, so the convergence speed and search ability are enhanced. Then, by constructing an industrial WSN model with scale-free characteristics, the FW-PSO algorithm is applied to the network topology optimization, and the dynamic and static invulnerabilities of the optimized network are analyzed through simulation experiments. The experimental results show that, no matter what the value of parameter α (the adjustable parameter of initial load strength) is adjusted, the network optimized by the algorithm proposed in this paper not only has the largest percentage reduction of the critical threshold *T_c_* under two kinds of attack strategies (HD and LD), and shows strong invulnerability, but also has the largest percentage improvement of the network connectivity under the random attack, and the connectivity is significantly enhanced. So the optimized network by FW-PSO algorithm has significant improvement in dynamic and static invulnerabilities than some other optimal algorithms, which indicates the feasibility and practicality of FW-PSO algorithm to optimize the network topology of industrial WSN.

The purpose of this paper is to explore how to construct the network topology with strong invulnerability, and it needs all the topological information of the network in the process. The influences of MAC (Media Access Control) layer of the network (such as transmission delay and packet loss rate) and energy factor on network performance are not considered too great. In addition, the topology we constructed is undirected graph, which does not sufficiently consider the flow direction of data in the network. In our next work, we would like to consider more practical factors to further improve our model.

## Figures and Tables

**Table 1:** Simulation parameters setting.

Items	Value
Node distribution area	100 × 100 m^2^
Number of network nodes (*popsize*)	100
Number of network edges (*W*)	191
Acceleration factor ($c_{1}$, $c_{2}$)	1.49445
Minimum inertia weight ($w_{\min}$)	0.4
Maximum inertia weight ($w_{\max}$)	0.9
Explosion number adjustment factor *A*	5
Explosion number adjustment factor *M*	6
Explosion number limit factor *a*	0.3
Explosion number limit factor *b*	0.6
Total number of iterations $gen_{\max}$	100
PSO iterations *maxgen*	300
